# Effect of UV-A, UV-B and UV-C irradiation of glyphosate on photolysis and mitigation of aquatic toxicity

**DOI:** 10.1038/s41598-020-76241-9

**Published:** 2020-11-20

**Authors:** Dimitra Papagiannaki, Claudio Medana, Rita Binetti, Paola Calza, Peter Roslev

**Affiliations:** 1 Società Metropolitana Acque Torino S.p.A.—Centro Ricerche, Torino, Italy; 2 Dipartimento di Biotechnologie Molecolari e Scienze della Salute, Università di Torino, Torino, Italy; 3 Dipartimento di Chimica, Università di Torino, Torino, Italy; 4 Department of Chemistry and Bioscience, Aalborg University, Aalborg, Denmark

**Keywords:** Environmental sciences, Environmental chemistry, Pollution remediation

## Abstract

The active herbicide ingredient glyphosate [*N*-(phosphonomethyl)glycine] is frequently detected as a contaminant in groundwater and surface waters. This study investigated effects of UV-A (365 nm), UV-B (302 nm) and UV-C (254 nm) irradiation of glyphosate in water on photolysis and toxicity to aquatic organisms from different trophic levels. A test battery with bacteria *(Bacillus subtilis, Aliivibrio fischeri)*, a green microalga (*Raphidocelis subcapitata),* and a crustacean *(Daphnia magna)* was used to assess biological effect of glyphosate and bioactive transformation products before and after UV irradiation (4.7–70 J/cm^2^). UV-C irradiation at 20 J/cm^2^ resulted in a 2–23-fold decrease in toxicity of glyphosate to aquatic test organisms. UV-B irradiation at 70 J/cm^2^ caused a twofold decrease whereas UV-A did not affect glyphosate toxicity at doses ≤ 70 J/cm^2^. UV-C irradiation of glyphosate in drinking water and groundwater with naturally occurring organic and inorganic constituents showed comparable or greater reduction in toxicity compared to irradiation in deionized water. High-resolution mass spectrometry analyses of samples after UV-C irradiation showed > 90% decreases in glyphosate concentrations and the presence of multiple transformation products. The study suggests that UV mediated indirect photolysis can decrease concentrations of glyphosate and generate less toxic products with decreased overall toxicity to aquatic organisms.

## Introduction

Active ingredients from pesticide formulations are among the most frequently detected organic micropollutants in aquatic environments. Glyphosate-based herbicides represent a major pesticide category that can contaminate groundwater and surface waters through multiple routes including spray drift, surface runoff and soil leaching^1–4^. *N*-(phosphonomethyl)glycine is the active ingredient in glyphosate-based herbicides and result in non-selective and broad-spectrum products for control of annual and per annual weeds. Glyphosate-based herbicides are popular in the domestic and agricultural sectors, and glyphosate is now considered the most frequently used agricultural chemical worldwide^1,3^. A global glyphosate application of about 700,000 tons per year has caused an ubiquitous environmental occurrence of this organophosphorus compound, and glyphosate has been characterized as a potential threat to humans and aquatic life because non-target organisms can be adversely affected^1,3–8^. The continued approval of glyphosate for domestic and agricultural use has therefore been debated^1,3,8,9^.

A particular concern in many countries is pesticide contamination of water resources for drinking water production. In Europe, the European Council Directive 98/83 (1998) concerning water quality for human consumption states that the regulatory limit for individual pesticides as well as their metabolites and transformation products is 0.1 µg/L. Water treatment can remove or attenuate pesticide concentrations in drinking water by processes such as microbial degradation, coagulation, sorption (e.g., activated carbon), and oxidation (e.g., ozonation). Photochemical processes involving ultraviolet radiation (UV) can facilitate degradation of pesticides via direct or indirect photolysis but UV doses used for traditional drinking water treatment (e.g., disinfection) are relatively low and rarely able to facilitate removal of micropollutants such as pesticides. Different additives (oxidants or catalysts) may be added to enhance the degradation processes mainly through formation of reactive oxidants. Examples of such water treatment processes are the advanced oxidation involving the addition of hydrogen peroxide (e.g., UV-C/H_2_O_2_) and photocatalysis involving the addition of different catalysts (e.g., UV-C/TiO_2_)^10–13^. In contrast, direct and indirect effects of elevated UV irradiation without added oxidants or catalysts for removal of pesticides from drinking water have received less attention. However, UV irradiation without additives could be attractive for drinking water treatment because the technique is non-invasive and free of added chemicals.

There are several potential mechanisms by which UV irradiation could mediate the transformation of pesticides in water^2^: (1) UV irradiation can cause direct photochemical transformation of a pesticide by absorption of photons followed by different chemical reactions such as bond cleavage and/or oxidation–reduction, (2) UV irradiation can interact with H_2_O and O_2_ and form reactive oxygen species including hydroxyl radicals (·OH) and hydrogen peroxide (H_2_O_2_) that interact with the pesticide and cause degradation. The outcome of such processes may have many practical implications but depends on several factors including the water matrix and the UV exposure regime (e.g., UV dose and UV wavelength). UV irradiation is electromagnetic radiations with different wavelengths (e.g., 10–400 nm) and can be divided into different categories such as UV-A, UV-B, UV-C and Vacuum UV. Only very few studies have considered the impact of different UV categories on the removal of glyphosate and glyphosate-based herbicides from water although some studies have suggested a potential of UV-C^14–16^. Hence, little is currently known about the direct and indirect effects of different types of UV irradiation of aqueous glyphosate on the removal of the parent compound and generation of bioactive transformation products. However, it appears highly relevant to assess if such UV transformation products are potentially more or less toxic than the parent compound and if there are differences among UV irradiation regimes.

In this study, we examined the effect of UV-A (365 nm), UV-B (302 nm) and UV-C (254 nm) irradiation of glyphosate in aqueous solutions on the occurrence of transformation products and the toxicity to aquatic test organisms. The major purpose was to determine if UV irradiation could decrease toxicity of glyphosate and to identify relevant UV exposure regimes (UV wavelength, UV dose). Changes in growth and activity of test organisms were compared before and after UV irradiation using a battery of non-target organisms that included *Bacillus subtilis, Aliivibrio fischeri*, *Raphidocelis subcapitata,* and *Daphnia magna.* Organisms from different trophic levels were used in the experiments to better assess the biological effects of all bioactive compounds in the samples after UV treatment including glyphosate transformation products. To the best of our knowledge, this is the first time the ecotoxicity of glyphosate to non-target organisms from different trophic levels has been compared before and after exposure to UV irradiation with different wavelengths (UV-A, UV-B, UV-C).

## Results

### Test organisms

**Figure 1:**
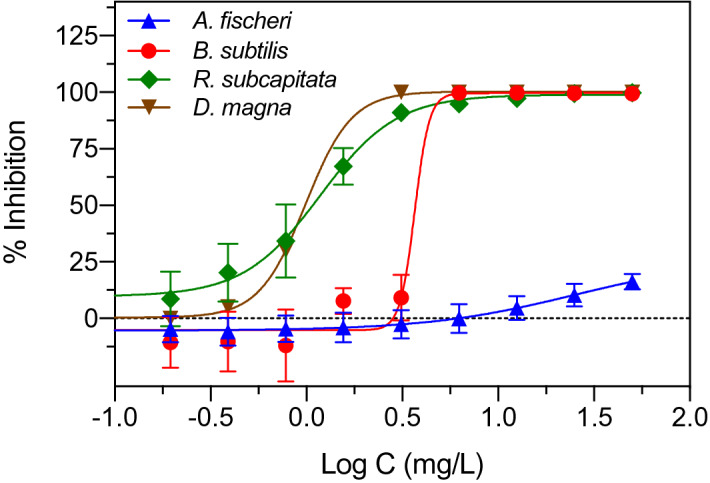
Toxicity of glyphosate to different aquatic test organisms measured as concentration–response curves. Data points represent means ± standard deviation.

Initial experiments were conducted to identify test organisms responsive to glyphosate exposure (Fig. 1). The traditional screening organism *A. fischeri* was the least responsive organism with an apparent median effective concentration (EC50) of 25.0 mg/L. In contrast, the crustacean *D. magna*, the bacterium *B. subtilis* and the green microalga *R. subcapitata* showed inhibition at much lower glyphosate exposure concentrations with EC50 values of 0.99 mg/L, 3.67 mg/L and 1.13 mg/L, respectively (Fig. 1). It was subsequently decided to focus on *D. magna, B. subtilis* and *R. subcapitata* for experiments examining changes in toxicity after UV irradiation of aqueous glyphosate. The selected test battery represented organisms from different trophic levels (bacteria, algae, zooplankton) to integrate the biological effect of known and unknown constituents in the samples after UV exposure including transformation products and oxygen radicals.

### Effect of UV irradiation on glyphosate toxicity

**Figure 2:**
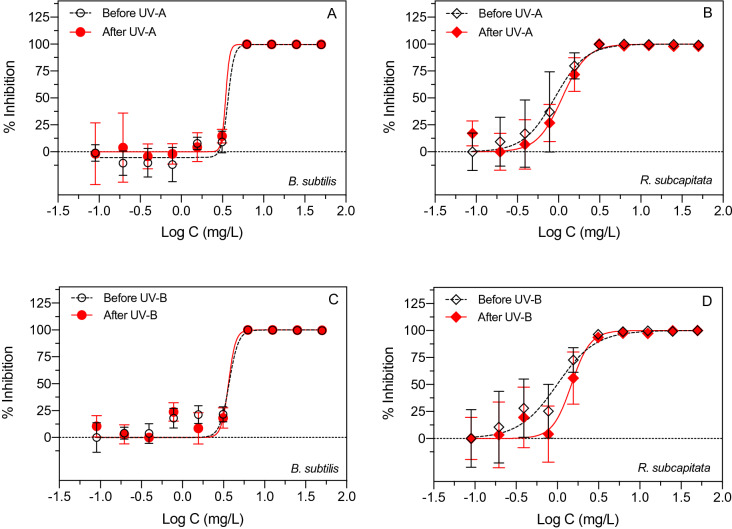
Effect of UV-A irradiation (**A**,**B**) and UV-B irradiation (**C**,**D**) of aqueous glyphosate (20 J/cm^2^) on toxicity to *B. subtilis* (**A**,**C**) and *R. subcapitata* (**B**,**D**). Data points represent means ± standard deviation.

**Figure 3:**
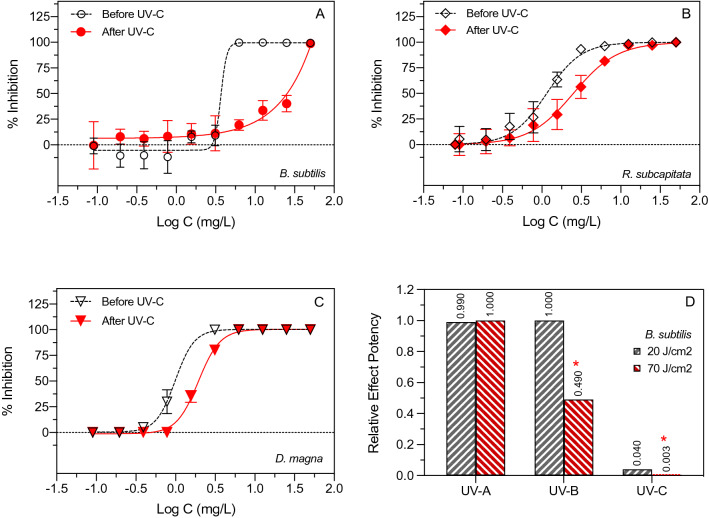
Effect of UV-C irradiation (**A**–**C**) of aqueous glyphosate (20 J/cm^2^) on toxicity to *B. subtilis* (**A**), *R. subcapitata* (**B**) and *D. magna* (**C**). Data points represent means ± standard deviation. Panel D shows the effect of an increased UV irradiation dose of 70 J/cm^2^ on the relative effect potency of glyphosate to *B. subtililis.* An asterisk (*) indicates a significant difference between 20 and 70 J/cm^2^ (Mann–Whitney, p < 0.05).

Toxicity of glyphosate before and after exposure to UV-A (365 nm), UV-B (302 nm) and UV-C (254 nm) irradiation was examined using *B. subtilis* and *R. subcapitata* and *D. magna* as test organisms (Figs. 2 and 3). Exposure of glyphosate to UV-A (20 J/cm^2^) and UV-B (20 J/cm^2^) did not have any noticeable effect on subsequent toxicity to the test organisms (Fig. 2A–D). In contrast, UV-C exposure (20 J/cm^2^) clearly decreased the toxicity of aqueous glyphosate to *B. subtilis*, *R. subcapitata* and *D. magna* (Fig. 3A–C).

**Figure 4:**
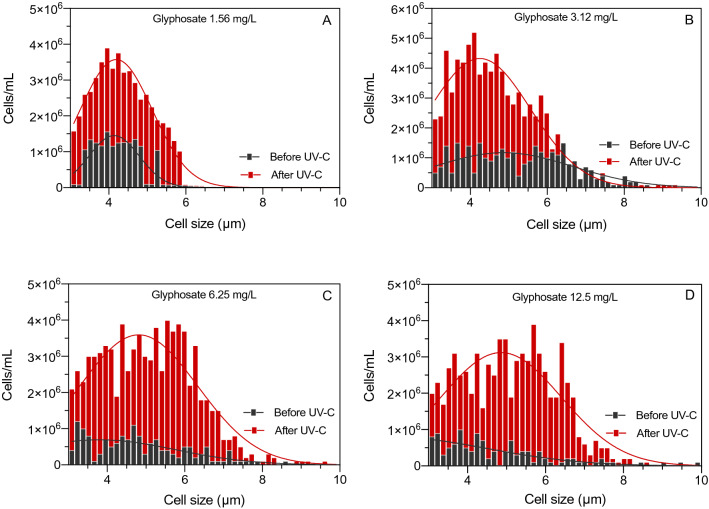
Effect of UV-C irradiation (20 J/cm^2^) of aqueous glyphosate on toxicity to *R. subcapitata* measured as differences in cell numbers and cell sizes after 72 h of growth in the presence of non-irradiated and irradiated glyphosate.

Changes in growth of *R. subcapitata* before and after UV-C treatment of glyphosate were measured as changes in absorbance as described in international standards (ISO 8692, 2012)^17^. The results were confirmed by counting and sizing individual algae cells using a Multisizer Coulter Counter (Fig. 4). Noticeable differences in cell numbers of *R. subcapitata* were observed after 72 h of growth in solutions with and without UV-C treatment of glyphosate (Fig. 4). For each of the four glyphosate concentrations, the difference between non-irradiated and UV-C irradiated solutions was significantly different as determined by the Mann–Whitney U test (p = 0.026, p < 0.001; p < 0.001; p < 0.001, respectively).

The median effective concentration of samples with glyphosate increased after UV-C irradiation (20 J/cm^2^) for all test organisms suggesting lower toxicity (Table 1). The UV-C irradiation caused a twofold, fivefold and 23-fold decrease in toxicity of glyphosate to *D. magna*, *R. subcapitata* and *B. subtilis* as indicated by the increases in EC50. A limited effect of UV on glyphosate toxicity was observed for UV-A and UV-B irradiation at 20 J/cm^2^ (Fig. 2A–D and Table 1). Increasing the UV-A dose to 70 J/cm^2^ did not change the toxicity of glyphosate to *B. subtilis* when estimated as relative effect potency (Fig. 3D). However, exposure of glyphosate to UV-B irradiation at 70 J/cm^2^ resulted in a significant decrease in toxicity relative to UV-B at 20 J/cm^2^ (Mann–Whitney; p = 0.028). Furthermore, increasing the UV-C dose from 20 J/cm^2^ to 70 J/cm^2^ also significantly decreased the toxicity of glyphosate (Mann–Whitney; p = 0.029). This suggests that UV-C and also UV-B exposure can decrease ecotoxicity of glyphosate if the UV dose is sufficiently high.

**Table 1:** Median effective concentrations (EC50) for three aquatic test organisms before and after exposure of glyphosate to UV-A, UV-B or UV-C at comparable UV doses (20 J/cm^2^). *ND: not determined.*

Test organism	EC50 values (mg/L)			
	Before UV	After UV-A	After UV-B	After UV-C
*B. subtilis*	3.67	3.45	3.54	85.41
*R. subcapitata*	1.13	1.19	1.53	5.70
*D. magna*	0.99	*ND*	*ND*	1.93

The presence of active oxygen species in the aqueous samples after UV irradiation was confirmed in experiments using different oxygen radical probes. Superoxide radical (·O_2_^−^), was confirmed as chemiluminescence after immediate injection of luminol into samples after UV treatment (posttreatment addition), whereas hydroxyl radical (·OH) formation was detected as increased fluorescence after the addition of coumarin, terephthalic acid and benzoic acid to aqueous samples before UV treatment (pretreatment addition).

### Effect of UV-C exposure regimes on glyphosate toxicity

**Figure 5:**
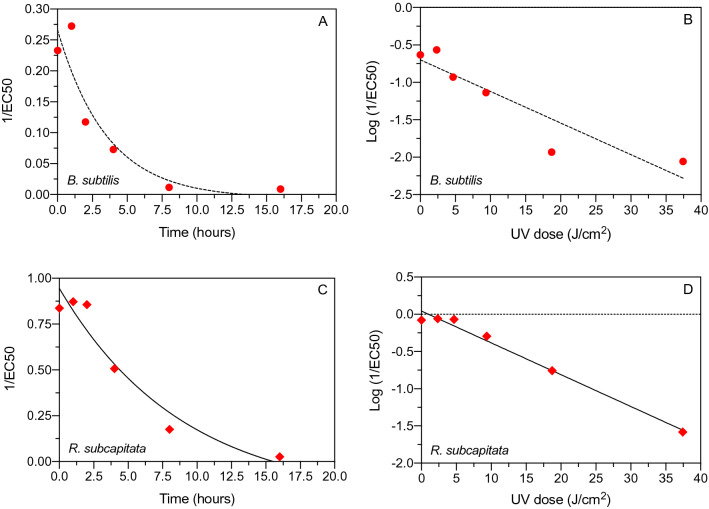
Effect of UV-C irradiation time (h) and UV-C dose (J/cm^2^) on toxicity of glyphosate to *B. subtilis* (**A**,**B**) and *R. subcapitata* (**C**,**D**).

The toxicity of glyphosate to *B. subtilis* and *R. subcapitata* decreased exponentially with increasing glyphosate irradiation time and dose (Fig. 5). The relationship between UV-C dose and decrease in toxicity, calculated as log(1/EC50), suggests a loss of 90% of the initial glyphosate toxicity (D90) to *B. subtilis* and *R. subcapitata* after UV-C irradiation corresponding to 23.4 J/cm^2^ and 23.7 J/cm^2^*,* respectively. Hence, toxicity was attenuated for both organisms in a UV-C dose-dependent relationship and with comparable rates suggesting that the test organisms were equally responsive to UV-C mitigation of glyphosate inhibition (Fig. 5).

Exposure of glyphosate to UV irradiation was mainly carried out using sealed quartz cuvettes with aqueous samples. It was subsequently examined if a different exposure method would result in comparable results. Experiments with different UV-C doses suggested that doses < 10 J/cm^2^ may affect toxicity (Fig. 5). Table 2 shows the results of an experiment where UV-C exposure in quartz cuvettes at 5.4 J/cm^2^ was compared with UV-C exposure in UV transparent plastic microplates at 5.4 J/cm^2^. The results did not indicate major differences in median effective concentration for the exposure regimes (quartz vs. transparent plastic), and the mitigation of glyphosate toxicity by UV-C appeared independent of the two exposure methods (Table 2).

**Table 2:** Median effective concentrations (EC50) for two aquatic test organisms before and after exposure of glyphosate to 5.4 J/cm^2^ UV-C.

Exposure technique	*B. subtilis* EC50 values (mg/L)		*R. subcapitata* EC50 values (mg/L)	
	Before UV-C	After UV-C	Before UV-C	After UV-C
Quartz Glass	3.67	7.92	1.13	4.18
UV plate	2.34	7.12	1.47	3.65

Two different UV exposure techniques were compared: UV-C exposure of glyphosate in quartz glass cuvettes and UV-C exposure in UV transparent plastic microplates.

All toxicity experiments in this study included control samples with UV exposure of aqueous samples without glyphosate to assess any toxicity associated with active oxygen species generated during the UV irradiation process. No apparent inhibition of test organisms was observed due to such products (Mann–Whitney U test, p > 0.05).

### Effect of UV irradiation on toxicity of glyphosate in drinking water

**Figure 6:**
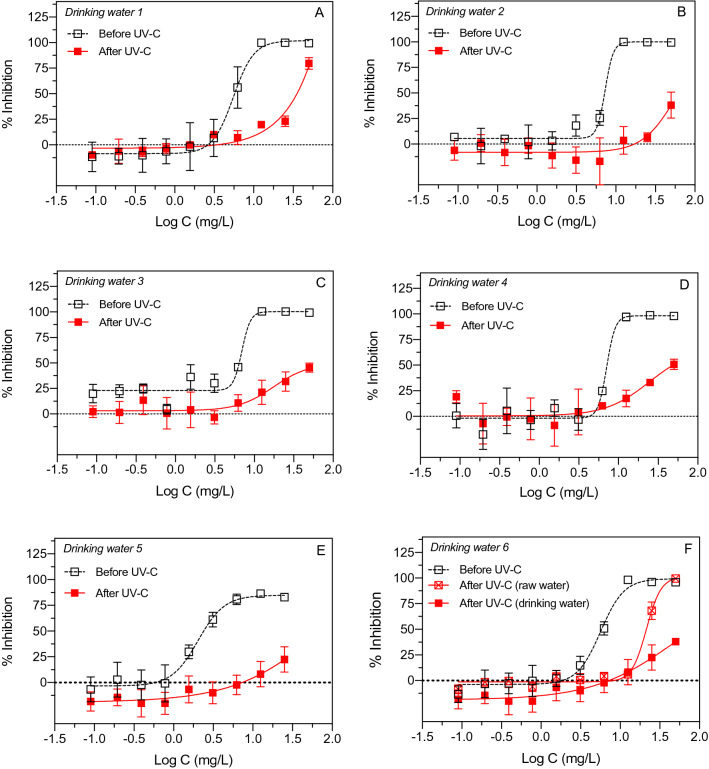
Effect of UV-C irradiation (20 J/cm^2^) of glyphosate in municipal drinking water on toxicity to *B. subtilis.* Drinking water produced from groundwater was collected at six locations in three Danish municipalities: Aalborg Municipality (**A**—Aalborg East; **B**—Aalborg Center; **C**—Aalborg West), Sønderborg Municipality (**D**), Aarhus Municipality (**E**), and Elsted drinking water treatment plant in Aarhus Municipality sampled before and after water treatment (**F**). Data points represent means ± standard deviation.

The potential for decreasing toxicity of aqueous glyphosate by UV irradiation was initially examined using a test matrix with deionized distilled water and artificial freshwater to minimize effects from unknown water constituents including organic and inorganic compounds. After some promising initial results with UV-C irradiation, experiments were subsequently conducted with natural drinking water samples obtained from 6 locations in Denmark. The drinking water samples consisted of municipal groundwater-based drinking water with variable concentrations of organic and inorganic constituents (https://eng.geus.dk/products-services-facilities/data-and-maps/national-well-database-jupiter/). Glyphosate spiked drinking water samples showed clear differences in toxicity before and after irradiation with 20 J/cm^2^ of UV-C (Fig. 6). In some cases, the decrease in toxicity due to UV-C treatment of aqueous glyphosate was slightly larger for the natural drinking water samples compared to parallel experiments conducted in distilled water (Fig. 3 vs. Fig. 6). The UV effect was also greater for glyphosate added to drinking water compared to groundwater (raw water) (Fig. 6F). The raw water was slightly colored and contained elevated concentrations of natural elements such as iron, manganese and ammonia because the water was sampled before filtration at the Drinking Water Treatment Plant. For glyphosate irradiated in drinking water, the EC50 values before UV-C varied between 2.03 mg/L and 7.30 mg/L whereas the EC values after UV-C varied between 17.57 mg/L and > 100 mg/L. The differences in EC50 before and after UV-C irradiation were significantly different (Mann–Whitney; p = 0.002). The Relative Effect Potency after UV-C treatment was 0.02–0.4 corresponding to a 3 to 44-fold reduction in toxicity to the test organism *B. subtilis*. Hence, the effect of UV-C on glyphosate was not inhibited by constituents in the drinking water samples and may even be more pronounced in some water matrices than in deionized water suggesting that natural drinking water may facilitate the process.

### Identification of glyphosate phototransformation products

Liquid chromatography high-resolution mass spectrometry analysis (LC-HRMS) of water samples with glyphosate was performed before and after UV-C irradiation to identify transformation products after UV doses of 20 J/cm^2^ and 70 J/cm^2^ (Table 3). The analysis was carried out as a semi-targeted analysis focusing on likely glyphosate transformation products using the METLIN database (https://metlin.scripps.edu/)^18,19^. The LC-HRMS analysis confirmed that aqueous concentrations of glyphosate decreased by 96% after UV-C irradiation and that degradation had occurred. More than 20 glyphosate (C_3_H_8_NO_5_P) transformation products were observed after UV-C irradiation (Table 3) including main products such as sarcosine (C_3_H_7_NO_2_), glycine (C_2_H_5_NO_2_), glyoxylic acid (C_2_H_2_O_3_), aminomethylphosphonic acid (CH_6_NO_3_P; AMPA), acetic acid (CH_4_O_2_) and phosphoric acid (H_3_PO_4_). CO_2_ was also a potential transformation product but was not targeted in the analysis due to a high background concentration of CO_2_/HCO_3_^−^ in the water samples. Increases in AMPA and glycine concentrations were observed after UV-C irradiation at 70 J/cm^2^, whereas sarcosine concentrations decreased. Phosphoric acid was detected at both high and low UV-C doses whereas glyoxylic and acetic acid were only observed after UV-C irradiation at 70 J/cm^2^. Glyphosate concentration decreased in samples irradiated with 20 J/cm^2^ and was no longer detectable in samples treated with 70 J/cm^2^.

**Table 3:** Identified transformation products by LC-HRMS analysis in negative ESI(-) and positive ESI(+) modes after UV-C irradiation of aqueous glyphosate.

Compound	*m/z*	RT (min)	ESI	Compound	m/z	RT (min)	ESI
H_3_O_4_P	96.968	25.94	−	C_5_H_9_NO_2_	116.069	15.66	+
CH_6_NO_3_P	110.001	1.75	−	C_3_H_3_NO_2_	84,009	27.82	−
C_2_H_5_NO_2_	74.020	1.86	−	C_3_H_4_O_4_	103.003	1.69	−
C_2_H_2_O_3_	72.908	16.64	−	C_3_H_6_O_4_	105.017	26.14	−
C_3_H_7_NO_2_	90.054	29.62	+	C_3_H_6_O	58.080	11.8	+
C_2_H_7_NO_2_	77.084	1.77	−	C_3_H_9_NO_2_	92.069	1.66	+
C_2_H_5_NO	59.070	35.32	−	C_4_H_10_O_2_	91.074	28.37	+
C_2_H_6_N_2_O_4_	91.016	28.99	−	C_4_H_8_O	73.028	4.43	+
C2H_4_O_2_	59.015	8.52	−	C_4_H_8_O_4_	119.033	6.85	−
C_3_H_8_N_2_O	89.070	30.13	+	C_4_H_10_O_3_	106.120	29.42	−
C_6_H_11_NO_2_	130.085	28.58	+	C_5_H_5_N	80.048	32.84	+

## Discussion

Targeted and non-targeted chemical analyses can provide valuable information about the presence and concentrations of pesticides and their tranformation products in aquatic systems. In vitro and In vivo bioassays can complement chemical analyses by providing information about the presence of bioactive compounds in a sample including bioactive transformation products^20^. This is relevant because traditional chemical analyses are often less suited for providing direct information about bioactivity and interactions among bioactive chemicals and transformation products. In the present study, we combined LC–HRMS analysis of glyphosate degradation in water with bioassays to assess removal and bioactivity before and after UV irradiation.

UV mediated photolysis of organic micropollutants can occur directly via photon absorption or indirectly in reactions mediated by active oxygen species^2,21–23^. The outcome of these processes depends on many factors including the structure and functional groups of the parent compound and the UV wavelength and dose. In some matrices, interactions between UV light and H_2_O and O_2_ can generate small amounts of photoproducts such as hydrogen peroxide (H_2_O_2_), hydroxyl radical (·OH) and superoxide anion (·O_2_^-^), and these active oxygen species can subsequently interact with dissolved or dispersed organic matter^21,24^. In the present study, LC–HRMS analysis confirmed that aqueous concentrations of glyphosate decreased after UV-B and UV-C irradiation and that degradation had occurred. Glyphosate degradation was non-linear with respect to irradiation time with relatively more glyphosate being removed initially. This is supported by a first-order decrease in toxicity with UV-C irradiation time, i.e., the greatest toxicity decrease occurred during the initial irradiation phase (Fig. 5A,B). A range of potentially bioactive transformation products were observed after UV-C irradiation of aqueous glyphosate such as sarcosine, glycine, glyoxylic acid, aminomethylphosphonic acid (AMPA), acetic acid and phosphoric acid (Table 3). These transformation products are comparable to those reported in studies employing different Advanced Oxidation Technologies for glyphosate removal^11–13,25,26^. Such studies have proposed several possible degradation mechanisms, focusing mainly on the cleavage of the N–C and P–C bonds. The results obtained in our study corroborate these pathways. The potential pathways for the UV mediated photolysis of glyphosate are shown in the Supplementary material (Fig. S1). The first pathway (“N–C pathway”) involves the breakdown of the N–C bond directly by UV or by oxidizing radicals generated in the water samples, resulting in the formation of AMPA and acetic acid or formation of glyoxylic acid. The second pathway (“P–C pathway”) involves the formation of phosphoric acid and sarcosine which after treatment with elevated UV dose is transformed into glycine. Both pathways shown in Fig. S1 are in accordance with previous studies and will result in less toxic transformation products thereby supporting the results of the toxicity tests.

A very limited number of studies have addressed the transformation of glyphosate in water with only UV irradiation without added oxidizing agents or catalysts. A previous study reported that glyphosate solution in deionized water (1 mg/L) decreased by 50% after 4 days of UV-C exposure at 20 °C and an UV intensity of 30 W/cm^2^^14^. A study by Bourgeois et al.^27^ suggested that degradation of a 300 mg/L glyphosate solution was possible using a low-pressure UV lamp at 254 nm and that the degradation was more efficient than using a medium-pressure lamp at 200-600 nm (UV dose not stated). Balah ^28^ stated that an aqueous solution of glyphosate at 30 μg/L decreased by 99% in concentration after 120 min exposure to UV irradiation at 254 nm–336 nm (UV dose not stated). Assalin et al.^15^ observed slow glyphosate degradation and formation of AMPA after UV-C irradiation of 42 mg/L glyphosate in water, and Sandy et al.^16^ observed generation of orthophosphate after UV mediated photooxidative degradation of glyphosate. Our results add to these findings and suggest that UV mediated photolysis can decrease glyphosate concentrations by > 90% in aqueous solutions and generate different transformation products. Even if the UV mediated photodegradation of glyphosate is incomplete, it may subsequently increase biodegradation in aquatic environments if the transformation products are less toxic and/or more biodegradable. The two main biodegradation pathways employed by glyphosate-degrading environmental microorganisms include cleavage of the C-N and the C-P bond converting glyphosate to AMPA and sarcosine^29^. Interestingly, AMPA and sarcosine were among the transformation products detected in the present study after UV-C irradiation of glyphosate. Although glyphosate is often considered less persistent in the environment compared to AMPA, this compound is nonetheless detected in many aquatic systems including groundwater and surface waters^1,3,30^. The results above suggest a potential for combining UV treatment with bioremediation to further increase remediation of glyphosate.

A measure of the effectiveness of UV irradiation as a mitigation method for glyphosate should include an assessment of the potential to alleviate toxicity to non-target organisms of the parent compound and the transformation products. Glyphosate can affect a range of non-target organisms including freshwater invertebrates, fish and amphibians ^1,31–38^. The effects of different glyphosate-based herbicides (commercial formulations) on non-target plants and algae have also been reported^38–44^. In the present study, the EC50 for *D. magna* exposed to glyphosate was 0.99 mg/L which is comparable or slightly lower than those reported in previous studies^35,36,45^. The EC50 values for the green algae *R. subcapitata* and the bacterium *B. subtilis* were 1.13 and 3.67 mg/L which is also in range reported in previous studies^1,38,40,41,43^. Hence, the test organisms applied in this study to indicate glyphosate toxicity before and after UV irradiation expressed responses comparable to other studies and appeared suitable to indicate toxicity to non-target organisms. Our results suggest that UV-C and to some extend UV-B mediated indirect photolysis of glyphosate in water can decrease overall toxicity to several non-target organisms by generating less toxic transformation products (e.g., acetic acid, glycine, sarcosine, AMPA). To the best of our knowledge, our study is one of the few indicating that UV-C and high UV-B doses can facilitate photolysis of glyphosate without addition of chemicals or catalysts. In this context, it should be noted that the UV doses applied in the present study (4680–70,000 mJ/cm^2^) is above those typically applied for disinfection of drinking water and wastewater (40–100 mJ/cm^2^) but in the same range as reported previously for degradation of organic micropollutants (> 1000 mJ/cm^2^)^7^.

In the present study, identification of transformation products and analysis of toxicity after UV irradiation was carried out using diluted stocks of pure *N*-(phosphonomethyl)glycine without any additives. However, commercial brands of glyphosate-based herbicides often contain various surfactants and formulating agents. Hence, we included a screening of selected commercial formulations for reduction of toxicity after UV-C exposure (Roundup ”Ready to Use” (Monsanto), Roundup Garden (Monsanto), Gallup Super 360 (Barclay), Glyfonova 450 Plus (FMC). The screening of the commercial glyphosate formulations also suggested a potential of UV-C mediated mitigation of ecotoxicity (data not shown). Hence, more studies are needed to examine direct and indirect photolysis of such complex formulation including characterization of transformation products.

The majority of glyphosate concentrations used in the present study (mg/L) are clearly above the levels typically measured in surface waters and drinking water, which are often in the ng/L to µg/L range^1,30^. As mentioned above, previous studies have shown that direct and indirect photolysis can effectively degrade organics at different target concentrations, and we suggest that this may also be the case for trace level concentrations of glyphosate. Nonetheless, further studies should focus on UV effects at lower glyphosate concentrations and the applicability for different water types and under different hydraulic conditions. Such studies may also include UV techniques such as Vacuum UV that will likely increase oxidation potential and contaminant removal^46,47^. Because the suggested UV based techniques are fundamentally non-invasive and free of added reactants, they may be attractive for various water treatment practices including drinking water treatment.

## Conclusions

Glyphosate is frequently detected as contaminant in water resources and there are on-going scientific and public discussions in many countries about the safety and continued use of glyphosate-based herbicides. This study investigated effects of UV-A, UV-B and UV-C irradiation of glyphosate on photolysis and toxicity to aquatic organisms from different trophic levels. The results suggested that UV-C and to some extend UV-B mediated indirect photolysis of glyphosate in water could attenuate concentrations of this pesticide and decrease overall ecotoxicity. UV-C mediated generation of less bioactive glyphosate transformation products may subsequently facilitate environmental biodegradation. The study also emphasized that toxicity assays represent important supplements to chemical tools for water quality assessment because bioassays can integrate overall changes in water chemistry and bioactivity before and after water treatment. The results of our study can be relevant for further developments of UV mediated treatment processes for aquatic contaminants.

## Methods

### Chemicals

Stock solutions of glyphosate were prepared in autoclaved distilled deionized water from a 40% wt/vol *N*-(phosphonomethyl)glycine, monoisopropylamine salt solution (CAS 38641-94-0, Sigma-Aldrich), and from *N*-(phosphonomethyl)glycine (CAS 1071-83-6, Sigma-Aldrich). Stock solutions of commercial brands of glyphosate-based herbicides were prepared in autoclaved distilled deionized water from Roundup Ready to Use containing 7.2% wt/vol glyphosate (Monsanto, USA), Roundup Garden containing 12% wt/vol glyphosate (Monsanto, USA), Gallup Super 360 containing 36% wt/vol glyphosate (Barclay, UK), and Glyfonova 450 Plus containing 45% wt/vol glyphosate (FMC, Denmark). According to the product datasheets, these formulations also contain water and various surfactants and formulating agents. All stock solutions of glyphosate and glyphosate-based herbicides were stored in the dark at 5 °C.

### Drinking water samples

Drinking water samples were collected at Aalborg, Aarhus, Skagen and Sønderborg municipalities (Denmark). Water samples were also collected at the influent (raw water) and effluent (treated water) of Elsted drinking water treatment plant (Denmark). All water samples originated from groundwater but with regional differences in organic and inorganic constituents according to the national Danish well database on water quality (https://eng.geus.dk/products-services-facilities/data-and-maps/national-well-database-jupiter).

### UV irradiation of glyphosate in aqueous samples

Glyphosate and glyphosate-based herbicides dissolved in distilled deionized water or drinking water were exposed to different doses of UV-A, UV-B or UV-C irradiation. Stock solutions with 50 and 100 mg/L glyphosate were exposed to UV irradiation at 22 °C in 10 mm 3.5 mL quartz cuvettes (Science Outlet Optical Quartz QS10 and Hellma Precision Quartz Suprasil QS10). Dilute glyphosate concentrations were prepared in UV bottom—transparent 96 well microplates (Nunc 96-well UV microplates, Thermo Scientific) to examine the effects of glyphosate concentrations between 0.18 and 100 mg/L on the outcome of UV irradiation. The UV microplates with different glyphosate concentrations were exposed from the top or bottom using similar UV doses as the quartz cuvettes with uniform glyphosate concentrations. The 96-well UV microplates were placed on a cooling plate to avoid heating and maintain the temperature at around 22 °C to minimize evaporation from the small sample volumes used in these plates (100 µL). In all UV experiments, parallel control samples without UV exposure were covered with aluminium foil and stored in the dark for later analysis together with UV exposed samples.

UV irradiation experiments were carried out using a 4 W UVP UVGL-25 lamp (Analytic Jena) equipped with separate UV-A (365 nm) and UV-C (254 nm) tubes, and an 8 W UVP 3UV lamp (Analytic Jena) equipped with separate tubes for UV-A (365 nm), UV-B (302 nm) and UV-C (254 nm). The two UV lamps gave comparable results in toxicity experiments for comparable UV doses, and the 8 W lamp with greater intensity was selected to shorten exposure times. Irradiation intensity was measured using an Extech SDL470 Light meter equipped with UV-AB and UV-C sensors. The irradiation intensity at 15 cm distance from the UVP 3UV lamp was 970 μW/cm^2^/s for UV-A, 1900 μW/cm^2^/s for UV-B, and 327 μW/cm^2^/s for UV-C. UV doses (J/cm^2^) were calculated from the measured UV irradiation intensity (μW/cm^2^/s) and the exposure time (s). No detectable UV-C irradiation was measured from the UV-A and UV-B lamps and vice versa. UV exposure doses (J/cm^2^) were controlled by changing exposure times and distances to the UV lamps. For example, an exposure UV dose of 20 J/cm^2^ was obtained using the same exposure time but different distances to the UV lamp (20 cm for UV-A, 35 cm for UV-B, and for 5 cm for UV-C). The presence of active oxygen species in aqueous samples after UV irradiation was confirmed by the addition of different oxygen radical probes. Superoxide radical (·O_2_^-^), was detected by measuring chemiluminescence after posttreatment addition of 1 mM luminol. Hydroxyl radical (·OH) was detected by measuring fluorescence after pretreatment addition of 1 mM coumarin, terephthalic acid or benzoic acid. Chemiluminescence and fluorescence originating from the oxygen radical probes after reaction with active oxygen species were quantified using a Victor X2 Multilabel Plate Reader (Perkin Elmer).

### Analysis of glyphosate phototransformation products

Liquid chromatography**-**high resolution mass spectrometry analysis (LC-HRMS) of water samples with glyphosate was performed before and after UV exposure to identify transformation products. Analyses were carried out using an Ultimate 3000 High-Pressure Liquid Chromatography coupled through an ESI source to an LTQ-Orbitrap mass spectrometer (Thermo Scientific). Chromatographic separation was achieved using a reversed-phase C18 column (Phenomenex Luna, 150 × 2 mm, 3 µm, 110 Å; Phenomenex, Italy) by injecting a 10 µL sample volume at a mobile phase consisted of a mixture of 0.1 mM Formic Acid (eluent A) and Acetonitrile (eluent B). The gradient profile started with 5% B, increased up to 100% B in 40 min and to 100% A in 10 min. Samples were ionized in both positive and negative ionization modes. The LC effluent was delivered to the ESI ion source using Nitrogen as sheath and auxiliary gas with the following parameters: sheath gas 34 arbitrary unit (arb), auxiliary gas 15 arb, capillary voltage 4.48 kV, and capillary temperature of 270 °C. Full mass spectra were acquired in positive ion mode with a resolution of 30.000. Data analysis was performed using the MZmine 2.53^48^ for peak alignment, peak grouping, background noise and retention time correction, and the METLIN database was used to identify the transformation products^18,19,48^.

### Toxicity test with the luminescent bacterium *Aliivibrio fischeri*

Toxicity screening of glyphosate samples was examined in a standard inhibition tests with the luminescent bacterium *Aliivibrio fischeri* (ISO 11348-1, 2009)^49^. *A. fischeri* DSM 7151 was incubated in white 96-well plates (CulturPlate, Perkin Elmer), and exposed to the following concentrations of glyphosate with or without prior UV irradiation: 0.098, 0.195, 0.39, 0.78, 1.56, 3.13, 6.25, 12.5, 25, 50 mg/L. Changes in bioluminescence were quantified after 30 min using a Victor X2 Multilabel Plate Reader (Perkin Elmer).

### Toxicity test with the bacterium *Bacillus subtilis*

The toxicity of glyphosate samples to Gram positive bacteria was examined in a newly developed inhibition test with *Bacillus subtilis*. The endpoint was inhibition of growth and hydrolase activity after 18 h. *Bacillus subtilis* DSM 10 from the German Collection of Microorganisms and Cell Cultures (DSMZ) was grown at 30 °C in Davis Minimal Broth (Sigma-Aldrich) supplemented with the following trace elements: 25 µM FeSO_4_, 0.5 µM ZnCl_2_, 0.5 µM Na_2_MoO_4_, 0.5 μM MnCl_2_, 0.5 μM H_3_BO_3_, 0.5 μM CoCl_2_, 0.5 μM NiCl_2_, 2.0 μM CuSO_4_. Serial dilutions of glyphosate were made in 96-well clear Nunclon microplates (Thermo Scientific) using 100 µL glyphosate stock solutions (100 mg/L) serially diluted in 100 µL autoclaved distilled water resulting in different glyphosate concentrations (twofold dilutions and 100 µL diluted sample in each well). After glyphosate dilution, 50 µL of 4 × strength Davis Minimal Broth was added to each well, followed by addition of 50 µL of diluted *B. subtilis* culture (1:1000 dilution in 0.9% NaCl). This resulted in a final liquid volume of 200 µL in each well and 10 different concentrations of glyphosate: 0.098, 0.195, 0.39, 0.78, 1.56, 3.13, 6.25, 12.5, 25, 50 mg/L. Four replicates were included for blanks (medium only), controls (no glyphosate), and each glyphosate concentration. Sealed plates were incubated with shaking at 250 rpm for 18 h at 30 °C on a PST-60HL-4 Plate Shaker Thermostat (Biosan). The absorbance at 620 nm was then measured for each well using a Thermo Multiskan Plate Reader (Thermo Scientific). Finally, hydrolase activity in *B. subtilis* was measured by adding 20 µL fluorescein diacetate stock solution (5 mM) to each well to obtain a final concentration of 5 µM. After 60 min incubation at 30 °C, fluorescence was quantified in each well using a Victor X2 Multilabel Plate Reader with a 485 nm excitation and 535 nm emission filter (Perkin Elmer).

### Toxicity test with the green microalga *Raphidocelis subcapitata*

The toxicity of glyphosate to phytoplankton was examined in inhibition tests with the unicellular green microalga *Raphidocelis subcapitata* (formerly *Selenastrum capricornutum* and *Pseudokirchneriella subcapitata*; ISO 8692, 2012)^17^. The endpoint was inhibition of growth measured after 72 h of incubation (ISO 8692, 2012)^17^. *R. subcapitata* (MicroBioTests Inc.) was cultivated in test medium at 23 ± 2 °C and continuous illumination at 6500 lx (ISO 8692, 2012)^17^. Diluted culture was exposed in 96-well clear Nunclon microplates (Thermo Scientific) to different concentrations of glyphosate with or without prior UV irradiation (0.098, 0.195, 0.39, 0.78, 1.56, 3.13, 6.25, 12.5, 25, 50 mg/L). Eight replicates were included for blanks (medium), controls (no glyphosate), and each glyphosate concentration. Plates were incubated for 72 h at 23 °C on a shaker at 70 rpm with continuous illumination (6500 lx). Growth was measured after 0, 24 h, 48 h and 72 h as absorbance at 450 nm using a Thermo Multiskan Plate Reader (Thermo Scientific). Growth measurements for selected samples was verified by measuring cell sizes (µm) and cell abundance (cells/mL) using a Multisizer 4e Coulter Counter (Beckman Coulter).

### Toxicity test with the crustacean *Daphnia magna*

The toxicity of glyphosate to zooplankton was examined in inhibition tests with the crustacean *D. magna* (ISO 6341, 2012)^50^. The toxicological endpoint was inhibition of mobility determined by visual inspection of the animals (ISO 6341, 2012)^50^. *D. magna* STRAUS was cultivated from a laboratory clone originating from pure-culture ephippia^36^. Each treatment consisted of 20 juvenile animals distributed among 4 glass vials with 5 animals and 10 mL freshwater medium in each vial. The mobility of each animal was determined after 24 h and 48 h (ISO 6341, 2012)^50^.

### UV irradiation experiments

The following UV irradiation experiments with glyphosate were conducted to examine photolysis and biotoxicity before and after exposure of samples to UV irradiation: a) effect of UV wavelength (UV-A, UV-B, UV-C); b) effect of UV dose (J/cm^2^); c) effect of glyphosate concentrations (mg/L); and d) importance of water matrix. UV dosing between 2.3 and 70 J/cm^2^ was examined and 20 J/cm^2^ was selected as the default value in comparative tests. The effect of glyphosate concentration on the outcome of UV irradiation was examined in parallel experiments with a) UV-C irradiation (20 J/cm^2^) of a single concentration of glyphosate (100 mg/L) followed by serial dilution of the sample into 10 glyphosate concentrations for toxicity testing; b) UV-C irradiation (20 J/cm^2^) of 10 glyphosate concentrations between 0.18 mg/L and 100 mg/L followed by twofold dilution and toxicity testing. The effect of different water types on the outcome of UV irradiation experiments was examined by comparing UV-C irradiation of glyphosate in deionized water with glyphosate in drinking water samples from 4 Danish municipalities (Aalborg, Aarhus, Skagen, and Sønderborg).

### Data analysis and statistics

The toxic response measured for all endpoints were expressed as inhibition (I) relative to control samples: *I* = 1 − *(R*_*i*_/*R*_*c*_*)*, where *R*_*i*_ and *R*_*c*_ are responses measured for inhibited and control samples, respectively. Control samples included water samples with UV exposure but without glyphosate to assess any toxicity associated with active oxygen species generated during irradiation. Concentration–response curves were fitted to a log-logistic model using iterative non-linear regression^20^:

1 $${\text{Response}} = 1/(1 + 10^{(\log \;EC50 - \log C)} *{\text{Slope}}$$

where C is the toxicant concentration (mg/L), EC50 is the median effective concentration (mg/L), and Slope is a model parameter representing the slope of the curve. Iterative non-linear regressions and calculation of 95% confidence limits for EC50 values were performed using Prism 8.0.1 (Graphpad Software). Relative Effect Potency (REP)^20^ was used to estimate the toxicity of a sample before and after UV treatment:

2 $${\text{REP = EC50}}_{{{\text{(before)}}}} {\text{/EC50}}_{{{\text{(after)}}}}$$

where EC50_(before)_ is the median effective concentration before treatment (mg/L) and EC50_(after)_ is the median effective concentration after UV irradiation (mg/L).

Statistical analyses of results were carried out using the nonparametric Mann–Whitney U test (Wilcoxon rank sum test) for evaluating differences between treatments with a significance level of p < 0.05 (KaleidaGraph 4.5.4; Synergy Software).

## Supplementary information

Supplementary Figure 1.
