# Supplementary material for: Effect of UV-A, UV-B and UV-C irradiation of glyphosate on photolysis and mitigation of aquatic toxicity
Source: Sci Rep. 2020 Nov 20;10:20247. doi: 10.1038/s41598-020-76241-9 (PMC7679408; doi:10.1038/s41598-020-76241-9)
Supplement: Supplementary file 1 — Supplementary Figure 1. [file 41598_2020_76241_MOESM1_ESM.docx]

**SUPPLEMENTARY MATERIAL FOR MANUSCRIPT:**

Effect of UV-A, UV-B and UV-C irradiation
of glyphosate on photolysis and mitigation of aquatic toxicity

Dimitra Papagiannaki^1^, Claudio Medana^2^, Rita Binetti^1^, Paola Calza^3^ and Peter Roslev^4^

^1^Società Metropolitana Acque Torino S.p.A.—Centro Ricerche, Torino, Italy

^2^Università di Torino, Dipartimento di Biotechnologie Molecolari e Scienze della Salute, Torino, Italy
^3^Università di Torino, Dipartimento di Chimica, Torino, Italy
^4^Aalborg University, Department of Chemistry and Bioscience, Aalborg, Denmark

*Corresponding author: Peter Roslev, Department of Chemistry and Bioscience, Aalborg University, Aalborg, Denmark. Email: [pr@bio.aau.dk](mailto:pr@bio.aau.dk)

**FIGURE S1**

**
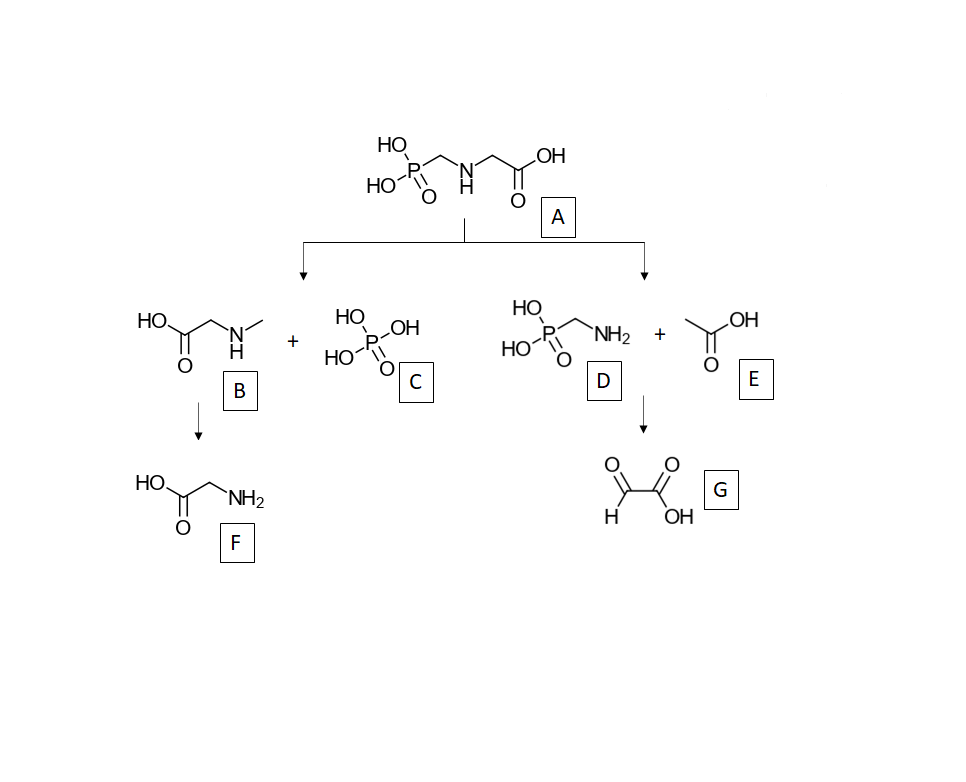
**

**Figure S1.** Potential pathways for UV mediated photolysis of Glyphosate (A) through two main routes resulting in sarcosine (B), and phosphoric acid (C), and AMPA (D) and acetic acid (E) or directly to glyoxylic acid (G). Sarcosine (B) may subsequently be transformed into glycine (F).
